# Fabrication and characterization of WO_3_/Ag/WO_3_ multilayer transparent anode with solution-processed WO_3_ for polymer light-emitting diodes

**DOI:** 10.1186/1556-276X-7-253

**Published:** 2012-05-15

**Authors:** Kangmin Jeon, Hongseok Youn, Seongbeom Kim, Seongbeom Shin, Minyang Yang

**Affiliations:** 1 Department of Mechanical Engineering, Korea Advanced Institute of Science and Technology, 373-1 Guseong-dong, Yuseong-gu, Deajeon, 305-701, South Korea

**Keywords:** Multilayer, Transparent anode, WO_3_, Polymer light-emitting diodes

## Abstract

The dielectric/metal/dielectric multilayer is suitable for a transparent electrode because of its high-optical and high-electrical properties; however, it is fabricated by an expensive and inefficient multistep vacuum process. We present a WO_3_/Ag/WO_3_ (WAW) multilayer transparent anode with solution-processed WO_3_ for polymer light-emitting diodes (PLEDs). This WAW multilayer not only has high transmittance and low resistance but also can be easily and rapidly fabricated. We devised a novel method to deposit a thin WO_3_ layer by a solution process in an air environment. A tungstic acid solution was prepared from an aqueous solution of Na_2_WO_4_ and then converted to WO_3_ nanoparticles (NPs) by a thermal treatment. Thin WO_3_ NP layers form WAW multilayer with a thermal-evaporated Ag layer, and they improve the transmittance of the WAW multilayer because of its high transmittance and refractive index. Moreover, the surface of the WO_3_ layer is homogeneous and flat with low roughness because of the WO_3_ NP generation from the tungstic acid solution without aggregation. We performed optical simulation and experiments, and the optimized WAW multilayer had a high transmittance of 85% with a sheet resistance of 4 Ω/sq. Finally, PLEDs based on the WAW multilayer anode achieved a maximum luminance of 35,550 cd/m^2^ at 8 V, and this result implies that the solution-processed WAW multilayer is appropriate for use as a transparent anode in PLEDs.

## Background

Recently, polymer light-emitting diodes (PLEDs) have been studied as next-generation light sources because they have high luminous efficiency and can be fabricated by an efficient solution process for high productivity [1-3]. However some layers of PELDs (the electron injection layer, cathode and anode) must be deposited by an expensive vacuum process; only the emitting and hole injection layers are coated by a solution process. Therefore, researchers have investigated extending the solution-processed layers [4-6]. The development of a solution-processed transparent electrode is particularly important because it is a fundamental component of organic electronics.

Indium tin oxide (ITO) is commonly used as the transparent anode for PLEDs because of its combination of high optical transmittance (>85% in the wavelength range of visible light) and low resistance (approximately 10 Ω/sq) [7]. However, ITO has some disadvantages. The supply of indium is constrained by both mining and geopolitical issues, and ITO must be deposited by a vacuum process that is expensive to set up and maintain. Therefore, researchers have investigated solution-processed transparent anodes such as conducting polymers and carbon nanotube films [8-12]. These anodes can be inexpensively coated by a solution process in an air environment, but their sheet resistances are ten times higher than that of ITO while their transmittance is similar [8,11]. Thus, conducting polymers and carbon nanotube films are unsuitable as transparent anodes for PLEDs.

Fan et al. reported dielectric/metal/dielectric (DMD) multilayers such as TiO_2_/Ag/TiO_2_[13], InZnO/Ag/InZnO [14], ZnS/Ag/ZnS [15-17], WO_3_/Ag/WO_3_ (WAW) [18], or ZnS/Ag/WO_3_[19,20] as transparent anodes. A DMD multilayer has low sheet resistance in the metal layer (approximately 5 Ω/sq) and high transmittance (>85%) because the refractive index discrepancy between the dielectric layers and the thin metal layer improves the transmittance of the metal layer [21]. However, it has low productivity because the vacuum process for a conventional DMD multilayer requires a high-degree vacuum for an extended time and has a limited chamber volume. This problem could be overcome by using a solution process. When dielectric layers are coated by a solution process, the productivity of the DMD multilayer is greatly improved, because the dielectrics form two layers in a DMD multilayer consisting of three layers. However, it is difficult to coat a thin and uniform dielectric layer using conventional sol-gel or nanoparticle (NP) solutions [22-24].

WO_3_ is one of the most suitable dielectric materials for the DMD multilayer. It has a high refractive index (*n* = 2.0 at wavelength of 580 nm) and high transmittance (>90% in the wavelength range of visible light) as well as high electrical conductivity [20]. However, in the conventional solution process WO_3_ has high surface roughness and too large particle size to form a dielectric layer thinner than 100 nm [25,26].

In this work, we develop a WO_3_/Ag/WO_3_ multilayer transparent anode with solution-processed WO_3_ for PLEDs. To coat thin WO_3_ layers by a solution process, we devise a novel method wherein WO_3_ NPs are synthesized from a precursor solution by rapid conversion that obstructs the growth of particles. Thin WO_3_ NP layers form WAW multilayer with a thermal evaporated Ag layer, and they improve the transmittance of the WAW multilayer without degradation of the Ag conductivity. The solution-processed WAW multilayer has excellent optical and electrical properties and higher productivity than the conventional multilayer because it can be fabricated by a high volume printing technologies. The optimal structure of the WAW multilayer is calculated by optical simulation, and the results are verified by comparison with experimental values. Finally, we evaluate the luminance of PLEDs based on the WAW multilayer transparent anode.

## Methods

### Fabrication of solution-processed WAW multilayer

**Figure 1:**
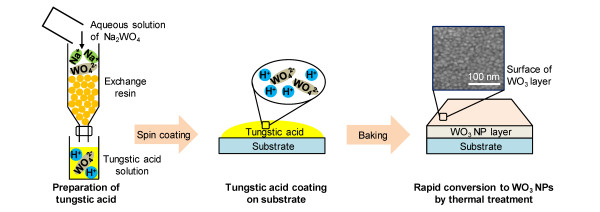
** Synthesis of WO**_**3**_**nanoparticles from aqueous solution of Na**_**2**_**WO**_**4**_**.** Surface of WO_3_ layer was investigated through field emission scanning electron microscope (FESEM).

**Figure 2:**
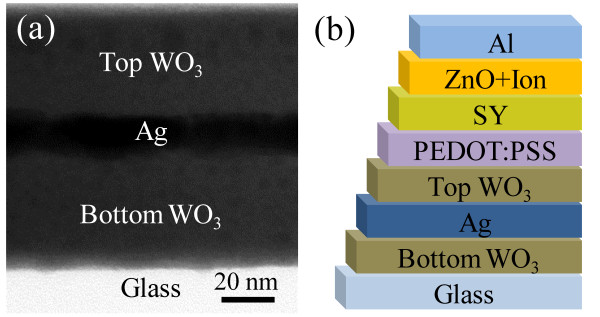
** Illustrations of WAW multilayer and WAW-based PLEDs.** (**a**) Cross-sectional transmission electron microscope (TEM) image of solution-processed WAW multilayer. (**b**) Schematic structure of WAW/PEDOT:PSS/Super Yellow/ZnO NPs and ionic interlayer/Al PLEDs.

To synthesize WO_3_, sodium tungstate (Na_2_WO_4_) was dissolved in deionized water without further purification. Its aqueous solution was passed at a fixed rate through a glass column packed with protonated cation exchange resin (TRILRTE SCR-BH), and the yellow effluent containing tungstic acid (H_2_WO_4_) was collected in a beaker, as shown in Figure 1[27,28]. The tungstic acid solution was mixed with isopropyl alcohol to improve the coating quality and then spin-coated on a glass substrate. The resulting thin layer was baked at 200°C to convert the tungstic acid to WO_3_. Subsequently, Ag (99.99%) was thermally evaporated on the WO_3_ layer in a high-vacuum chamber with a base pressure of 2 × 10^−6^ Torr. Finally, the tungstic acid solution was spin-coated on the Ag layer again and baked to complete the WAW multilayer as shown in Figure 2a. The thicknesses of the layers were measured by a surface profiler (Ahpha-Step 500, KLA-Tencor, Milpitas, CA, USA). The sheet resistance and optical transmittance were measured by the four-point probe technique (CMT-SR2000N, AIT, Gyeongi, South Korea) and a UV-VIS-NIR spectrophotometer (V-570, JASCO Inc., Easton, MD, USA), respectively.

### Fabrication of PLEDs on WAW multilayer anode

The PLEDs on the WAW multilayer anode were composed of layers of glass, the WAW multilayer, poly(3,4-ethylenedioxythiophene) poly(styrenesulfonate) (50 nm, PEDOT:PSS; Clevios P VP AI4083, Heraeus Precious Metal, Hanau, Germany), yellow light-emitting polymer (60 nm, ‘super yellow’ (SY), Merck & Co., Inc., Whitehouse Station, NJ, USA), ZnO NPs and ionic interlayer (20 nm, polyethylene oxide + tetra-n-butylammonium tetrafluoroborate), and Al, as shown in Figure 2b. The layers of WO_3_, PEDOT:PSS, super yellow, and ZnO NPs and the ionic interlayer were all fabricated by spin-coating, whereas the Ag and Al electrodes were thermally evaporated. PLEDs on a conventional ITO anode were fabricated as control devices for comparison. The resulting active area of each PLED is approximately 4.4 mm^2^. After the fabrication, the devices were measured without further encapsulations in air. The device characteristics of the current density, voltage, and luminance (J-V-L) curves were recorded with a source-measure unit (Keithley 2400, Keithley Instruments Inc., Cleveland, OH, USA) and a calibrated photodiode (CS-100A, Konica Minolta Optics, Inc., Tokyo, Japan).

## Results and discussion

### Preparation of solution-processed WO_3_

We passed an aqueous solution of Na_2_WO_4_ through a protonated cation exchange resin. The Na^+^ ions were replaced with H^+^ ions by the exchange resin, and we collected tungstic acid consisting of H^+^ and WO_4_^2−^ ions. The tungstic acid was converted to WO_3_ ·2H_2_O as a primary product, and then WO_3_ ·2H_2_O was calcined at 200°C for conversion to WO_3_ as a secondary product [28].

**Figure 3:**
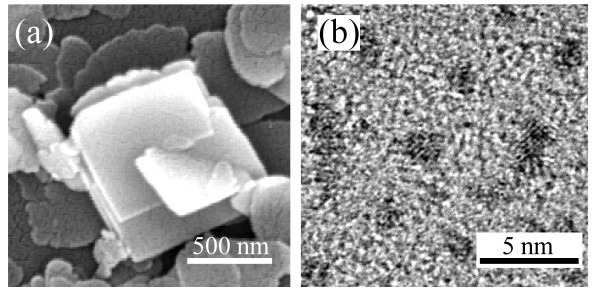
** Comparison of WO**_**3**_**particles.** (**a**) FESEM image of WO_3_ particles from conventional synthesis method. (**b**) TEM image of WO_3_ NPs less than 2 nm in size by the rapid conversion.

The WO_3_ ·2H_2_O particles were slowly synthesized from tungstic acid at room temperature, and the slow synthesis increased the particle size. Hence, the WO_3_ derived from these WO_3_ ·2H_2_O particles had irregular square plates with widths of 500 to 1,000 nm and thicknesses of 200 to 500 nm. Figure 3a shows a FESEM image of the WO_3_ particles. These large WO_3_ particles cannot form thin dielectric layers; a DMD multilayer is generally required to be thinner than 100 nm. To form a thin WO_3_ layer, we devised a new method that synthesized tiny WO_3_ NPs. The tungstic acid solution was coated on a glass and then baked at 200°C for rapid conversion and calcination. The rapid conversion obstructed the growth of WO_3_ ·2H_2_O particles, and WO_3_ NPs were synthesized from the WO_3_ ·2H_2_O particles after a calcination process. Figure 3b shows a TEM image of WO_3_ NPs less than 2 nm in size synthesized by the new synthesis method. Because these WO_3_ NPs are sufficiently small, the thickness of the WO_3_ layer can be controlled by adjusting the concentration and the coating condition of the tungstic acid solution in an air environment. Therefore, these WO_3_ NPs can form a thin dielectric layer to optimize the optical properties of the WAW multilayer.

**Figure 4:**
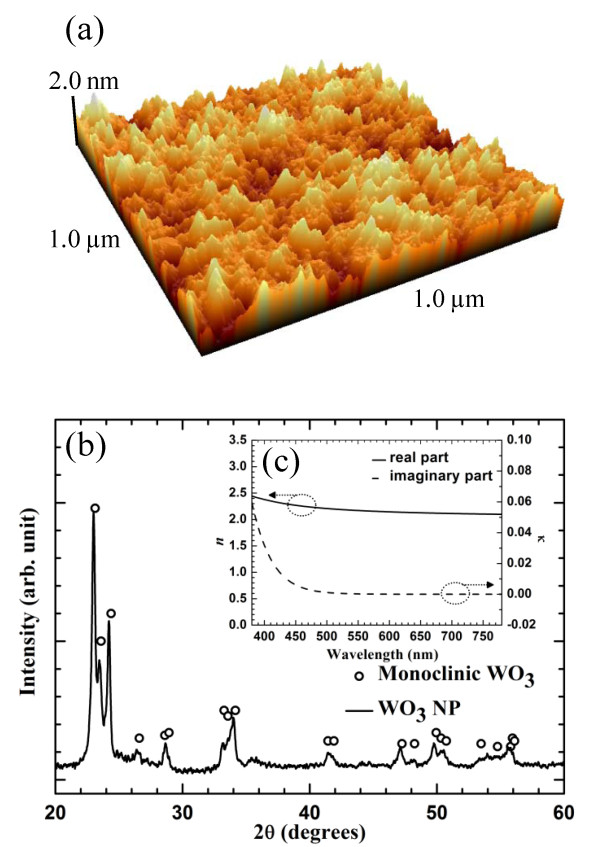
** Properties of WO**_**3**_** layer.** (**a**) Atomic force microscope (AFM) image of solution-processed WO_3_ layer surface. (**b**) Comparison of XRD patterns of WO_3_ NPs synthesized from an aqueous solution of Na_2_WO_4_ and monoclinic phase of WO_3_. (**c**) Real part (*n*) and imaginary part (*k*) of complex refractive index of WO_3_ layer.

This method has another advantage. Because the WO_4_^2−^ ions in tungstic acid were completely dissolved in deionized water, this aqueous solution could coat a uniform thin layer on the substrate without aggregation. The morphology of the WO_3_ layer was investigated by atomic force microscope (AFM; XE-100, Park Systems Corporation, Suwon, South Korea). Figure 4a shows that the surface of the WO_3_ layer was homogeneous and flat, with a low roughness (root mean square (rms) roughness = 0.457 nm, peak-to-valley roughness = 3.778 nm]. This WO_3_ layer with excellent surface quality can form WAW multilayer which has lower surface roughness than conventional transparent anode.

To verify the conversion of WO_3_ ·2H_2_O to WO_3_, we observed the crystallization of the WO_3_ NPs using an X-ray diffractometer (RIGAKU D/MAX-2500, Rigaku Corporation, Tokyo Japan). Solution-processed WO_3_ should be pure without WO_3_ ·2H_2_O. WO_3_ ·2H_2_O reduces the hole and the electron mobility of the dielectric layer of the WAW multilayer anode as impurity, and the low hole and electron mobility cause high turn-on voltage of PLEDs [16]. Figure 4b shows the XRD pattern of the solution-processed WO_3_. The typical sharp peaks of the monoclinic phase of WO_3_ are recognizable in the XRD pattern. This suggests that most of the WO_3_ ·2H_2_O is converted to WO_3_, and solution-processed WO_3_ is formed as a crystalline phase. Figure 4c shows the real part (*n*) and imaginary part (*k*) of complex refractive indices of the solution-processed WO_3_ layer. A 45-nm thick WO_3_ layer has higher refractive index than 2.0 in the wavelength range of visible light as in the case of a WO_3_ layer deposited using the evaporation process [19].

### Properties of WAW multilayer

We fabricated a WAW multilayer with solution-processed WO_3_ and investigated its optical and electrical properties as a transparent electrode. The transmittance of the multilayer is a function of the thicknesses and refractive indices of the materials [21]. Since the refractive indices of WO_3_ and Ag are fixed, the transmittance is determined by the thicknesses of the layers. It has been reported that a Ag layer has optimal electrical and optical properties when its thickness is 15 nm [14,16,19]. Therefore, the transmittance of the WAW multilayer should be optimized by controlling the thickness of the bottom and top WO_3_ layers.

**Figure 5:**
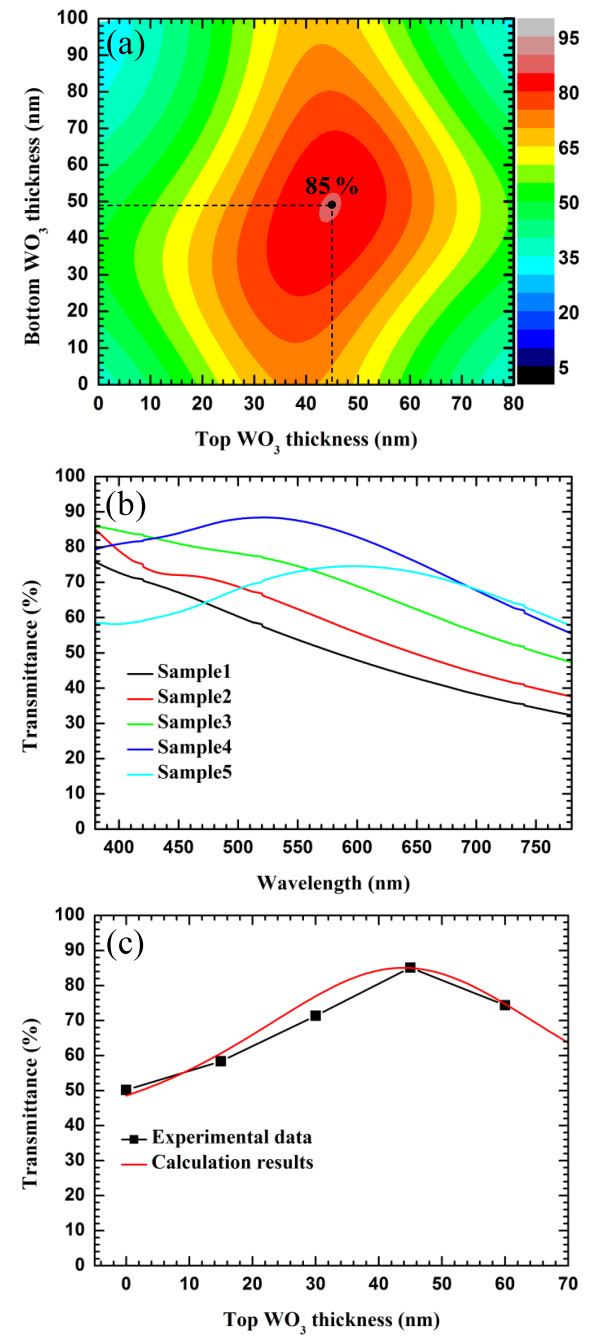
** Transmittance of WAW multilayer.** (**a**) Calculated transmittance of WAW multilayers at wavelength of 580 nm for different thicknesses of bottom and top WO_3_ layers. (**b**) Transmittance spectra of WAW multilayers of Table 1 in visible range. (**c**) Transmittance variation of calculated and experimental results at wavelength of 580 nm.

To optimize the structure of the WAW multilayer, we calculated its transmittance by a transfer matrix method. Figure 5a shows the calculated transmittance at a wavelength of 580 nm for different thicknesses of the bottom and top WO_3_ layers. The WAW multilayer had a maximum transmittance of 85% when the bottom and top WO_3_ layers were 49 and 45 nm, respectively. Table 1 shows the measured sheet resistance and transmittance of the Ag, ITO, and WAW multilayers. As the thickness of the top WO_3_ layer increased from 0 to 60 nm with the thickness of the bottom WO_3_ layer kept constant, the optical properties of samples 1 to 5 changed; however, all the samples had the same sheet resistance. These results indicate that the WO_3_ layers improve the transmittance without decreasing the Ag conductivity by the tungstic acid solution. Figure 5b shows the transmittance spectra of the samples of Table 1 in the visible range, and Figure 5c shows the transmittance variation of the calculated and experimental results at a wavelength of 580 nm. Sample 4 with WO_3_ (45 nm)/Ag (15 nm)/WO_3_ (45 nm) had the best transmittance (85%). This result indicates that the experimental transmittance is consistent with the calculated result.

**Table 1:** Transmittance and sheet resistance of Ag, ITO, and WAW multilayers.

**Number**	**Conditions**	**Transmittance (% at 580 nm)**	**Sheet resistance (Ω/sq)**
1	Ag 15 nm	50	approximately 4
2	WO_3_ 45 nm/Ag 15 nm/WO_3_ 15 nm	58	approximately 4
3	WO_3_ 45 nm/Ag 15 nm/WO_3_ 30 nm	71	approximately 4
4	WO_3_ 45 nm/Ag 15 nm/WO_3_ 45 nm	85	approximately 4
5	WO_3_ 45 nm/Ag 15 nm/WO_3_ 60 nm	74	approximately 4
6	ITO 150 nm	94	approximately 13

**Figure 6:**
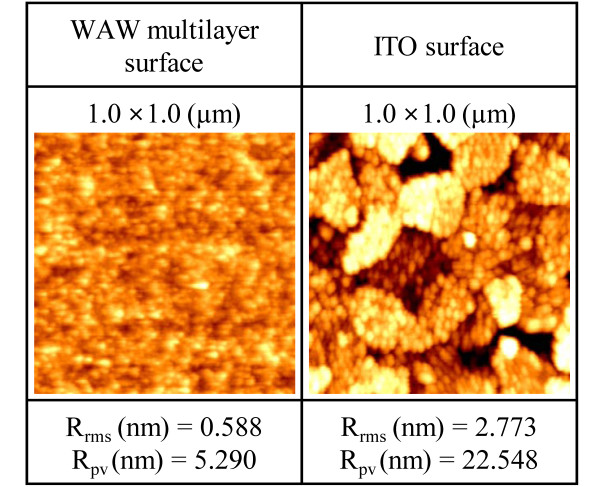
** Comparison of morphologies of WAW multilayer and ITO.** AFM image and surface roughness (root mean square and peak-to-valley) of solution-processed WAW multilayer and conventional ITO. R_rms_, rms surface roughness; R_pv_, peak-to-valley roughness.

Figure 6 shows the morphologies of the WAW multilayer and ITO measured by AFM. The rms surface roughness and peak-to-valley roughness of the WAW multilayer were 0.588 and 5.290 nm, respectively, and those of ITO were 2.773 and 22.548 nm, respectively. The WAW multilayer had a homogeneous and flat surface which is quite smooth compared to the surface of ITO. The surface roughness of the WAW multilayer anode is an important factor limiting the performance of PLEDs because thin layers of PEDOT:PSS and a light-emitting polymer (SY) must be in direct contact on the anode [6].

Consequently, the solution-processed WAW multilayer had a sheet resistance of 4 Ω/sq, an rms of 0.588 nm, and a transmittance of 85%, which is considerably high as compared to the 50% transmittance of a Ag layer. As a transparent anode for PLEDs, the solution-processed WAW multilayer is superior to ITO. This is because the WAW multilayer has better electrical conductivity and surface roughness than ITO, although its transmittance is slightly lower.

### Performance of PLEDs based on solution-processed WAW anodes

**Figure 7:**
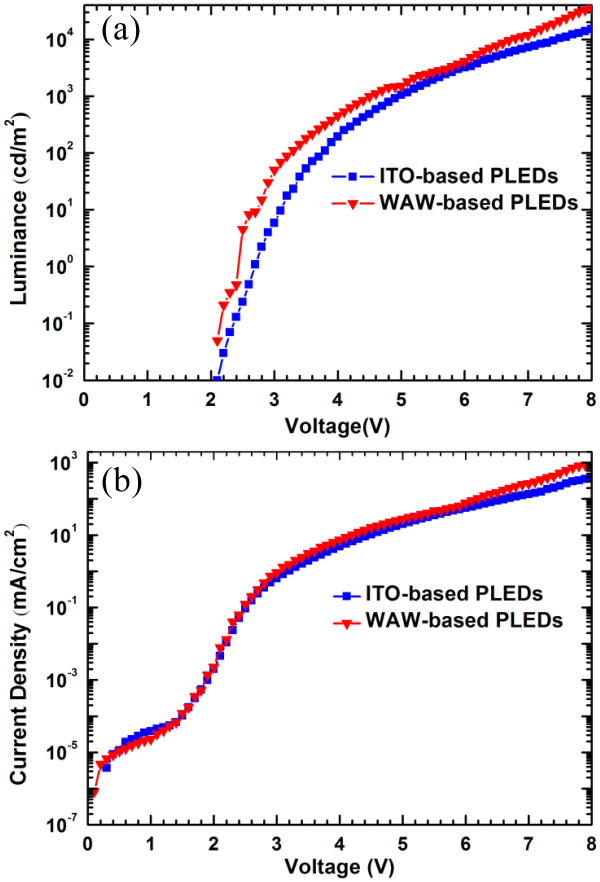
** Luminance characteristics of PLEDs.** Comparison of (**a**) luminance-voltage (L–V) and (**b**) current density-voltage (J–V) characteristics of ITO-based and WAW-based PLEDs.

**Figure 8:**
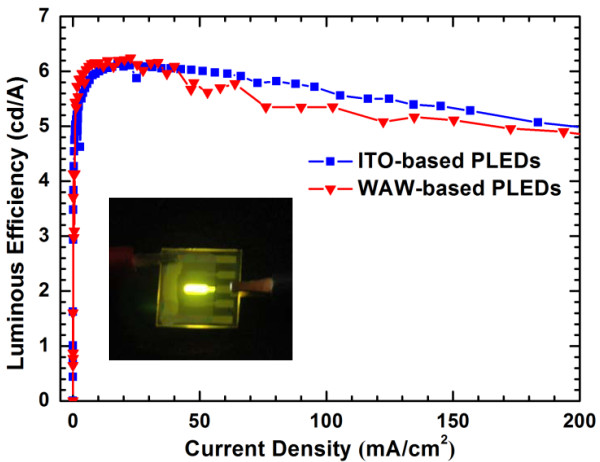
** Comparison of luminous efficiency of ITO-based and WAW-based PLEDs.** Inset shows photograph of WAW-based PLED in operation.

To evaluate the electrical and optical properties of the WAW multilayer as a transparent anode, we fabricated PLEDs based on the optimized WAW multilayer anode and the conventional ITO anode. The layer structure of the PLEDs was as follows: WAW multilayer/PEDOT:PSS/Super Yellow/ZnO NPs and ionic interlayer/Al. Figure 7 shows J-V-L characteristics of the WAW-based and ITO-based PLEDs. The turn-on voltages were identical (2.1 V). This implies that the hole-injection behavior of the solution-processed WAW multilayer was as good as that of ITO with the vacuum process because the turn-on voltage is one of the factors measuring the hole-injection property of an electrode [15,20]. The maximum luminance of the WAW-based PLEDs reached 35,550 cd/m^2^ at 8 V, whereas ITO-based PLEDs had a maximum luminance of 15,095 cd/m^2^ at 8 V. The maximum luminous efficiency of the WAW-based PLEDs (6.24 cd/A) was also higher than that of the ITO-based PLEDs (6.10 cd/A) as shown in Figure 8. These results indicate that the solution-processed WAW multilayer transparent anode is superior to ITO anode. The high conductivity of the WAW multilayer and the good contact quality of the WAW/PEDOT:PSS interface resulting from the low surface roughness of the WAW multilayer improve the performance of PLEDs [14].

## Conclusions

We have demonstrated the applicability of a highly productive WAW multilayer anode formed by a solution process as an alternative to conventional ITO anodes for PLEDs. To coat a thin and uniform WO_3_ layer by a solution process, we devised a new method in which WO_3_ NPs less than 2 nm in size were synthesized from an aqueous solution of Na_2_WO_4_. We then analyzed the crystallization characteristics of the WO_3_ NPs and the morphology of the WO_3_ NP layer. The solution-processed WAW multilayer, which was optimized via a simulations and experiments, had a sheet resistance of 4 Ω/sq and a transmittance of 85% at a wavelength of 580 nm. Moreover, WAW-based PLEDs had a maximum luminance of 35,550 cd/m^2^ at 8 V and a luminous efficiency of 6.24 cd/A. They were superior to ITO-based PLEDs because of the high conductivity of the WAW multilayer and the good contact quality of the WAW/PEDOT:PSS interface. Therefore, a solution-processed WAW multilayer is appropriate for both large-area displays and lighting applications because of its high productivity and excellent performance as a transparent electrode.

## Competing interests

The authors declare that they have no competing interests.

## Authors’ contributions

KJ participated in the fabrication of WAW multilayer and WAW-based PLEDs, and the analysis of their characteristics. HY participated in the analysis of current density, voltage, and luminance characteristics for PLEDs. SK and SS were involved in the FESEM, TEM, and XRD analysis of WO_3_ NPs and WAW multilayer. MY is the thesis director. All authors read and approved the final manuscript.
